# Human Sarcopenic Myoblasts Can Be Rescued by Pharmacological Reactivation of HIF-1α

**DOI:** 10.3390/ijms23137114

**Published:** 2022-06-26

**Authors:** Federica Cirillo, Laura Mangiavini, Paolo La Rocca, Marco Piccoli, Andrea Ghiroldi, Paola Rota, Adriana Tarantino, Barbara Canciani, Simona Coviello, Carmelo Messina, Giuseppe Ciconte, Carlo Pappone, Giuseppe Maria Peretti, Luigi Anastasia

**Affiliations:** 1 Laboratory of Stem Cells for Tissue Engineering, IRCCS Policlinico San Donato, Piazza Malan 2, 20097 San Donato Milanese, Italy; federica.cirillo@grupposandonato.it (F.C.); marco.piccoli@grupposandonato.it (M.P.); andrea.ghiroldi@grupposandonato.it (A.G.); tarantino.adriana@hsr.it (A.T.); simona.coviello@grupposandonato.it (S.C.); 2 Institute for Molecular and Translational Cardiology (IMTC), 20097 San Donato Milanese, Italy; paolo.larocca@unimi.it (P.L.R.); paola.rota@unimi.it (P.R.); giuseppe.ciconte@grupposandonato.it (G.C.); carlo.pappone@grupposandonato.it (C.P.); 3 Department of Biomedical Sciences for Health, University of Milan, Via Mangiagalli 31, 20133 Milan, Italy; laura.mangiavini@unimi.it (L.M.); carmelo.messina@unimi.it (C.M.); giuseppe.peretti@unimi.it (G.M.P.); 4 IRCCS Istituto Ortopedico Galeazzi, 20100 Milan, Italy; barbara.canciani@grupposandonato.it; 5 Department of Biomedical, Surgical and Dental Sciences, University of Milan, 20133 Milan, Italy; 6 Faculty of Medicine and Surgery, University Vita-Salute San Raffaele, Via Olgettina 58, 20097 Milan, Italy; 7 Arrhythmology Department, IRCCS Policlinico San Donato, Piazza Malan 2, 20097 San Donato Milanese, Italy

**Keywords:** HIF-1α, sarcopenia, atrophy, hypoxia, satellite cells

## Abstract

Sarcopenia, an age-related decline in muscle mass and strength, is associated with metabolic disease and increased risk of cardiovascular morbidity and mortality. It is associated with decreased tissue vascularization and muscle atrophy. In this work, we investigated the role of the hypoxia inducible factor HIF-1α in sarcopenia. To this end, we obtained skeletal muscle biopsies from elderly sarcopenic patients and compared them with those from young individuals. We found a decrease in the expression of HIF-1α and its target genes in sarcopenia, as well as of *PAX7*, the major stem cell marker of satellite cells, whereas the atrophy marker MURF1 was increased. We also isolated satellite cells from muscle biopsies and cultured them in vitro. We found that a pharmacological activation of HIF-1α and its target genes caused a reduction in skeletal muscle atrophy and activation of *PAX7* gene expression. In conclusion, in this work we found that HIF-1α plays a role in sarcopenia and is involved in satellite cell homeostasis. These results support further studies to test whether pharmacological reactivation of HIF-1α could prevent and counteract sarcopenia.

## 1. Introduction

The global increase in life expectancy is leading to a lasting change in the age structure of the population. The proportion of the world’s population that is 65 years or older is projected to increase from 9.3 percent in 2020 to 16.0 percent in 2050 [1]. The immediate consequence of this change is an increase in all age-related diseases, leading to an increase in the overall cost to public health systems. To reduce the impact on the public health system, many studies in the last decade have attempted to develop new approaches aimed at both preventing the occurrence of sarcopenia and finding new therapeutic interventions to reduce disability in the elderly [2,3].

Sarcopenia is the age-related loss of skeletal muscle mass associated with decreased muscle strength or physical function that negatively affects health [4]. It leads to impaired physical performance in the elderly, which is strongly associated with cardiovascular diseases (CVDs) [5]. Aging and skeletal muscle dysfunction limit physical performance in the elderly and increase the risk of CVDs [5]. CVDs and sarcopenia may occur simultaneously, further limiting physical performance, quality of life and increasing mortality [5]. They interact to accelerate the disease process [6,7]. As the world population ages, the prevalence of sarcopenia and CVDs is gradually increasing, which has significant implications for the health care system [4].

Sarcopenia has been shown to be characterized by a reduction in muscle fiber size, which is also fiber type specific, with a 10–40% reduction in the size of II type muscle fibers observed in muscle tissue of older individuals, compared to young controls [8]. Reduction of muscle fibers has been shown to be accompanied by an increase in interstitial non-contractile structures, such as fat and connective tissue [9]. Moreover, a reduction in mitochondrial density has been shown in sarcopenic muscle [10]. Tissue-level oxygen availability has been shown to change during aging, which is associated with alterations in hypoxia-inducible factor subunit alpha (HIF-1α) [11]. HIF-1α is the master regulator of oxygen-dependent expression of several target genes involved in oxygen transport, metabolic adaptation, new blood vessel formation, and various cellular functions, such as cell cycle regulation and apoptosis [12,13]. In aged mice, a decrease in binding to HIF-DNA was found to correlate with decreased expression of HIF-1α [14]. HIF-1α protein expression also decreases in smooth muscle cells isolated from rabbit aorta, rat cerebral cortex, carotid artery and mouse heart [14,15,16,17]. With respect to muscle mass, it was found that a decrease in HIF-1α expression in satellite cells of skeletal muscle from aged rats was directly associated with a decrease in angiogenesis and a loss of regenerative capacity [15].

Based on these premises, in this work, we further investigated the role of HIF-1α in sarcopenia by studying human skeletal muscle biopsies from elderly patients affected by sarcopenia and comparing them with those from healthy individuals. In addition, HIF-1α was pharmacologically reactivated in primary cultures of human satellite cells from sarcopenic patients to evaluate its effects on counteracting muscle wasting.

## 2. Results

### 2.1. Study Population and Clinical Characterization of the Human Sarcopenic Phenotype

**Figure 1:**
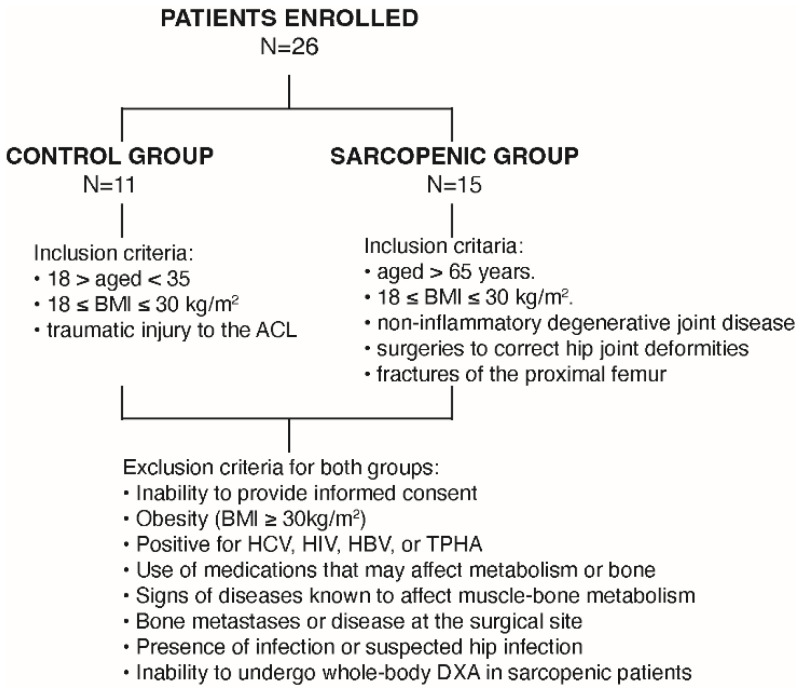
Flow chart of the study. Schematic representation of patient enrollment following the inclusion and exclusion criteria.

A total of 26 patients (15 males, 57%) were enrolled in this study. The sarcopenic group included 15 patients (mean age 82.9 ± 5.1 years), whereas 11 non-sarcopenic patients (mean age 30.8 ± 4.2 years) served as the control group (CTRL). Among the enrolled patients, the CTRL group was programmed for anterior cruciate ligament reconstruction, whereas the sarcopenic group was scheduled for hip replacement, due to primary or secondary osteoarthritis (Figure 1). 

**Figure 2:**
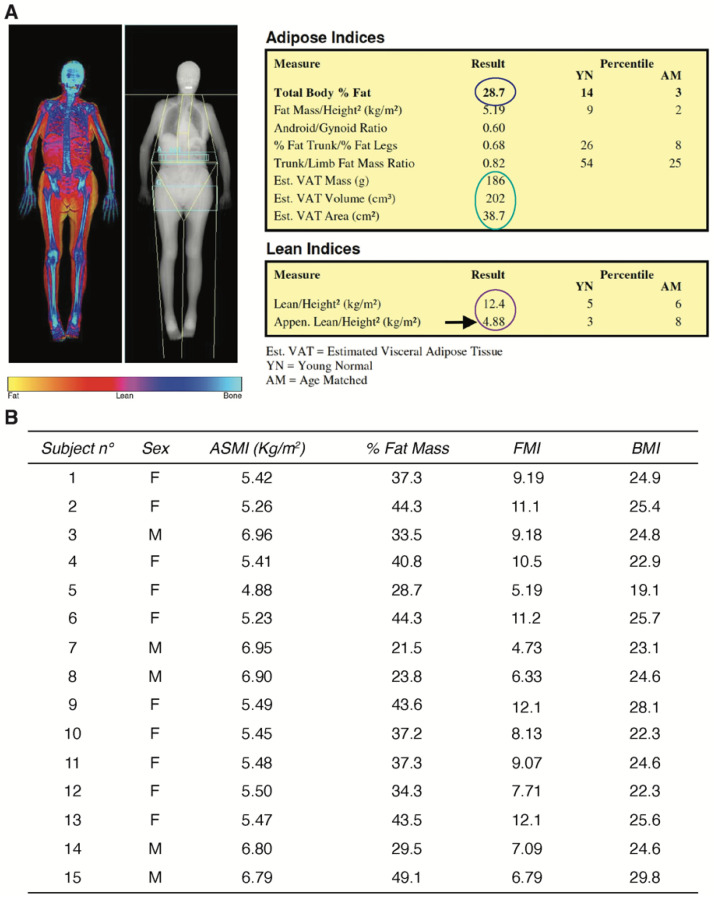
Dual-energy X-ray absorptiometry (DXA) imaging. (**A**) Representative whole-body image of sarcopenic female patients. The table on the right shows the percentage of total body fat (blue circle), visceral adipose tissue parameters (green circle), and parameters useful for assessing loss of muscle mass (purple circle); (**B**) results of body composition assessment of all sarcopenic patients who participated in the study: ASMI (appendicular skeletal muscle index for body size), FMI (fat mass index), BMI (body mass index).

Quantitative evaluation of fat and lean mass by Dual-energy X-ray absorptiometry (DXA) revealed appendicular skeletal muscle index (ASMI) and Fat Mass Index (FMI) values that indicated densitometrically reduced lean mass and confirmed the sarcopenic phenotype of the elderly patients (Figure 2A,B). 

**Figure 3:**
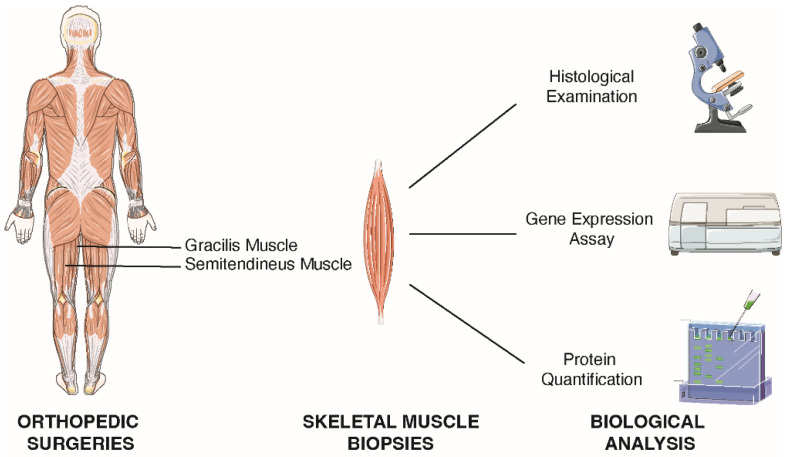
Schematic representation of the sampling collection and processing steps. Skeletal muscle biopsies of gracilis and semitendineus muscles were obtained during orthopedic surgery and characterized by histologic examination, gene expression, and protein analyzes.

A muscle biopsy was harvested from these patients during hip replacement surgery and further analyzed (Figure 3). As controls, muscle fragments were collected from young patients undergoing anterior cruciate ligament (ACL) reconstruction surgery. All patients tested negative for HCV, HBV, HIV, TPHA, and had no blood test alteration that might have affected muscle metabolism (data not shown).

### 2.2. Sarcopenia Induces the Activation of MURF1 and Reduction of PAX7 in Human Biopsies

**Figure 4:**
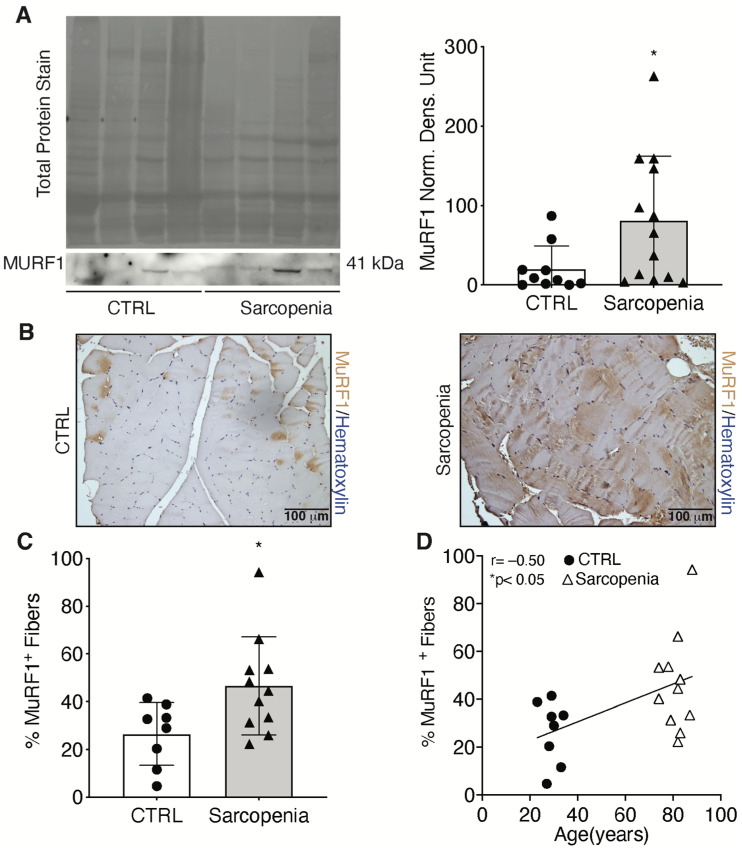
Modulation of MURF1 during sarcopenia. (**A**) Western blot analysis and relative quantification of MURF1 in CTRL and sarcopenic patients; (**B**,**C**) Immunohistochemical detection and relative quantification of MURF1 in CTRL and sarcopenic patients; (**D**) Correlation between MURF1 and patients’ age. Scale bar: 100 μm. Statistical significance was determined by the nonparametric Mann-Whitney test and by Pearson correlation coefficients. * *p* < 0.05.

Skeletal muscle atrophy has also been characterized at the molecular level by examining the alteration of MURF1 expression to confirm the clinical evaluation of CTRL and sarcopenic patients [18]. Specifically, skeletal muscle biopsies were used for RNA and protein extraction. No significant changes in mRNA expression of *MURF1* were detected, while protein levels were increased 4.3-fold in sarcopenic patients, compared with the CTRL group (Supplementary Figure S1; Figure 4A). In addition, the expression of MURF1 was also examined directly on human skeletal muscle biopsies (Figure 4B). Staining of MURF1 was performed on tissue sections, which showed a 1.7-fold increase confirming the atrophic phenotype of the sarcopenic patients compared with the CTRL group (Figure 4C). A positive significant correlation was found between MURF1 and the age of the patients (Figure 4D). 

**Figure 5:**
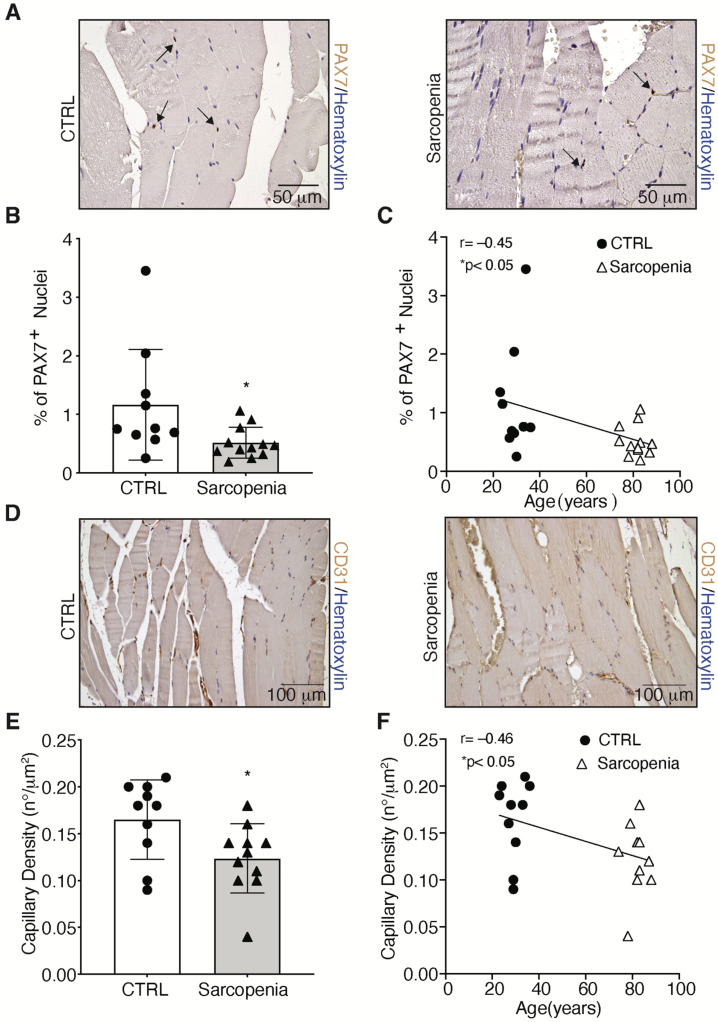
Impairment of skeletal muscle regeneration process in sarcopenia. (**A**,**B**) Immunohistochemical detection and relative quantification of PAX7-positive cells, indicated by black arrows, in CTRL and sarcopenic patients; (**C**) Correlation between PAX7 and patients’ age; (**D**,**E**) Immunohistochemical detection and relative quantification of CD31 in CTRL and sarcopenic patients; (**F**) Correlation between CD31 and patients’ age. Scale bar: PAX7 = 50 μm, CD31 = 100 μm. Statistical significance was determined by the nonparametric Mann-Whitney test and by Pearson correlation coefficients. * *p* < 0.05.

To further clarify the role of satellite cells (SCs) in sarcopenia, tissue sections were labeled with the marker for SCs, PAX7 to measure changes in patients as compared to controls (Figure 5A) [19]. Results showed that the number of SCs was reduced by 64% in skeletal muscle biopsies isolated from sarcopenic patients, as compared with controls (Figure 5B). Moreover, a negative correlation was found between the number of SCs and the age of the patients (Figure 5C). As muscle fiber capillarization is a marker of sarcopenia during aging [20], the number of capillaries in skeletal muscle biopsies from sarcopenic and CTRL patients was studied using CD31, the marker for endothelial cells [21]. Results showed that capillaries decreased by 25% compared to CTRL (Figure 5D,E). Moreover, the percentage of CD31 in the skeletal muscle tissue of the patients correlated negatively with age (Figure 5F).

### 2.3. The Hypoxic Signaling Pathway Is Strongly Impaired in Human Sarcopenic Patients

**Figure 6:**
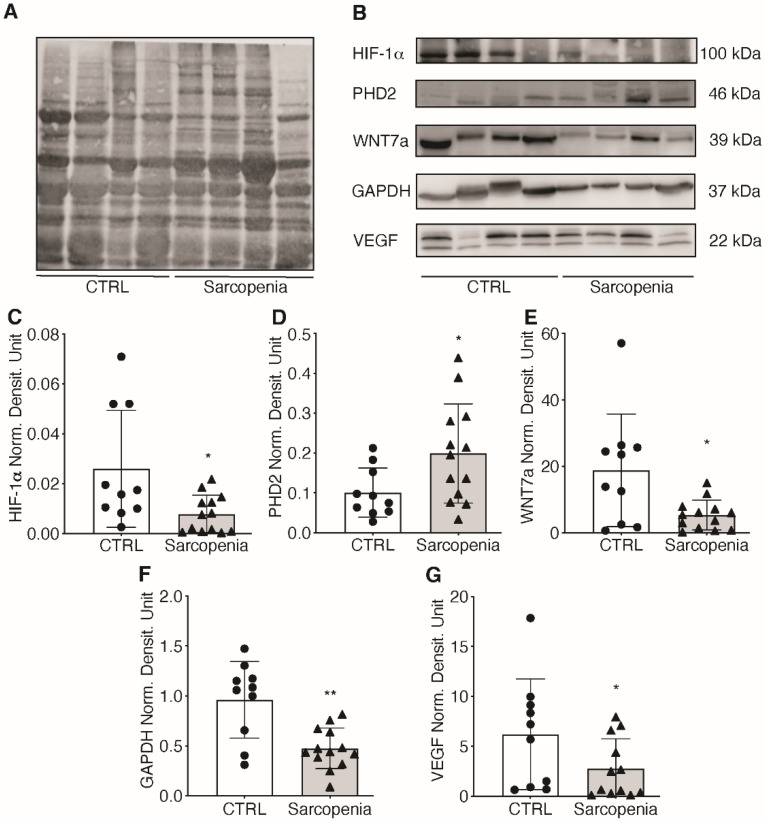
Impairment of HIF-1α in sarcopenic patients. (**A**) Total proteins of CTRL and sarcopenic patients were stained with Total Revert Protein Solution; (**B**) Western Blot of HIF-1α and its target genes: PHD2, WNT7a, GAPDH, and VEGF; (**C**–**G**) Relative quantification of HIF-1α activation pathway. Statistical significance was determined by the nonparametric Mann-Whitney test. * *p* < 0.05, ** *p* < 0.01.

As hypoxia is known to trigger neo-angiogenesis [22], changes in the hypoxic signaling pathway were investigated. The main focus was on HIF-1α, the master regulator of cell response to changes in oxygen tension. Total proteins were extracted from skeletal muscle biopsies from CTRL and sarcopenic patients and the activation of the HIF-1α signaling pathway was investigated (Figure 6A). Results showed that HIF-1α protein level decreased by 64% in sarcopenic patients compared with CTRL (Figure 6B,C). In addition, some of its target genes were examined to demonstrate the inactivation of the HIF-1α pathway. First, protein levels of prolyl hydroxylases 2 (PHD2), the enzymes responsible for HIF-1α hydroxylation and subsequent degradation, were analyzed [23]. The data showed a 2-fold increase in the amount of PHD2 in sarcopenic patients, which correlated with a reduction in HIF-1α (Figure 6B,D). WNT7a, a glycoprotein associated with skeletal muscle hypertrophy, and glyceraldehyde-3-phosphate dehydrogenase (GAPDH), an enzyme of the glycolytic pathway, showed a 73 and 57% reduction, respectively, in sarcopenic patients, as compared with CTRL (Figure 6B,E,F) [24,25,26,27]. In addition, a 62% decrease in VEGF content was observed, confirming the impairment of the vascular endothelial system (Figure 6B,G) [28]. 

**Figure 7:**
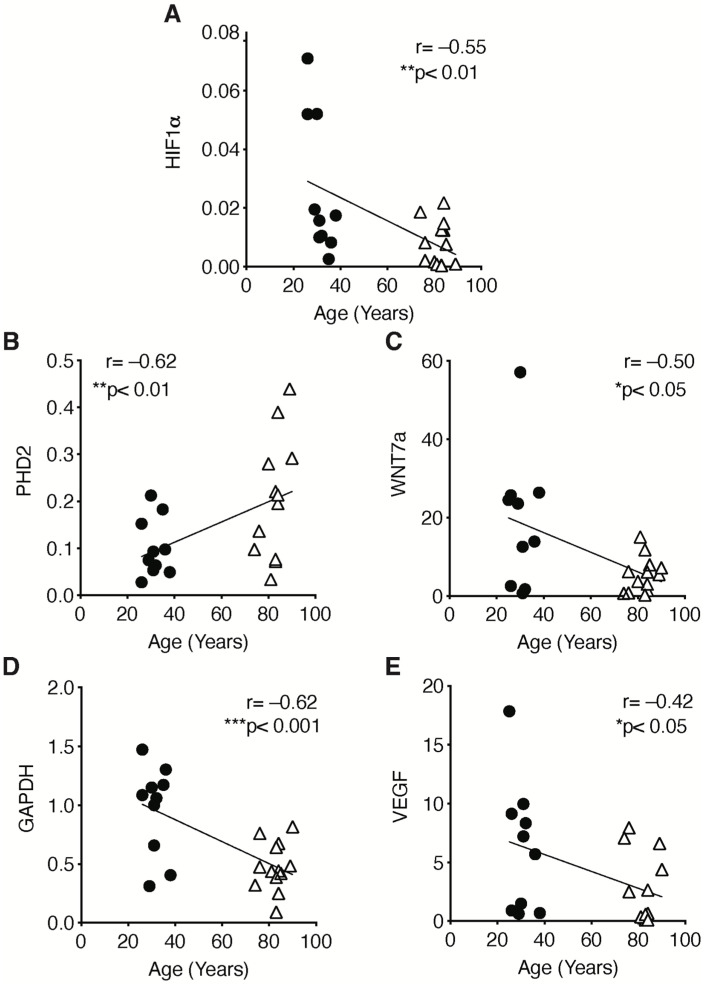
Correlation of HIF-1α signaling pathway and patients’ age. Correlation of (**A**) HIF-1α, (**B**) PHD2, (**C**) WNT7a, (**D**) GAPDH, (**E**) VEGF during aging. Statistical significance was determined by Pearson correlation coefficients. * *p* < 0.05, ** *p* < 0.01, *** *p* < 0.001.

To assess whether the impairment of HIF-1α signaling was associated with sarcopenia, the correlation between its expression (and that of its target genes) with the onset of the atrophic phenotype was examined. The results revealed a statistically negative correlation between HIF-1α and all its target genes (WNT7a, GADPH, and VEGF), with the exception of its inhibitor PHD2 (Figure 7A–E).

### 2.4. The Pharmacological Activation of HIF-1α Counteracts the Sarcopenic Phenotype

**Figure 8:**
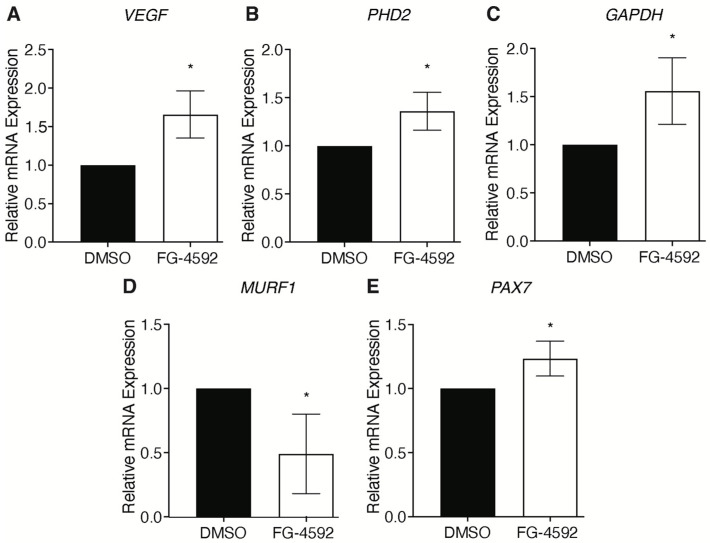
Effects of a pharmacological activation of HIF-1α on skeletal muscle atrophy. Gene expression of HIF-1α target genes in skeletal muscle cells treated with 50 μM FG-4592 for 24 h: (**A**) VEFG, (**B**) PHD2, (**C**) GAPDH. Modulation of (**D**) MURF1, and (**E**) PAX7 upon FG-4592 treatment. Statistical significance was determined by *t*-test. * *p* < 0.05.

To test whether HIF-1α activation would affect sarcopenia by preventing the onset of symptoms, human skeletal muscle cells from sarcopenic patients were isolated and treated with 50 μM FG-4592, a PHDs inhibitor, for 24 h [29]. To quantify the reactivation of the HIF-1α pathway, quantitative real-time PCR was performed on human skeletal muscle cells treated with vehicle (DMSO) or FG-4592. The results showed that treatment with FG-4592 triggers a 1.64-, 1.35-, and 1.55-fold increase in gene expression of *VEGF*, *PHD2*, and *GAPDH*, respectively, confirming the activation of the HIF-1α pathway (Figure 8A–C). Next, the effects of treatment with FG-4592 on the progression of skeletal muscle atrophy and on the impairment of the regeneration process were examined by analyzing *MURF1* and *PAX7*, respectively. Data showed that the reactivation of the HIF-1α pathway in human skeletal muscle cells isolated from sarcopenic patients resulted in a 50% decrease in atrophy marker *MURF1* gene expression (Figure 8D). Along this line, the improvement in muscle well-being was also reflected in a 1.25-fold increase in *PAX7* gene expression, supporting the restoration of the regenerative machinery (Figure 8E).

## 3. Discussion

To investigate the role of HIF-1α in sarcopenia, we first determined the atrophic phenotype of skeletal muscle biopsies from our sarcopenic patient cohort compared with biopsies from young individuals who served as controls. Results showed ASMI and FMI values indicative of densitometrically decreased lean mass, as well as a dramatic increase in MURF1, the major marker of skeletal muscle atrophy [18]. We also confirmed an age-dependent decrease in HIF-1α expression and its major target genes, VEGF and GADPH, whereas an upregulation of its inhibitor PHD2 was observed [23,25,28]. It has been reported that activation of HIF-1α promotes neo-angiogenesis and cell survival in various pathologies [30]. Furthermore, we recently discovered that HIF-1α also plays an important role in myogenesis [31]. Namely, we found that its action is due to direct binding to the promoter region of WNT7a, activating MYOD, the master regulator of skeletal muscle differentiation [24]. Moreover, pharmacological activation of HIF-1α induced the formation of hypertrophic myofibers in vitro and in vivo, whereas inhibition of the pathway impaired this process [24]. In this context, the atrophic phenotype observed in sarcopenia might be related to an impairment of the regenerative system. The loss of muscle mass seems to be associated with a reduced number of satellite cells (SCs), especially in fibers expressing myosin heavy chain type II (fast), or with an increased number of senescent SCs [32,33,34]. However, some studies have found no loss of SCs during aging in mouse and rat models [35,36]. Although the involvement of SCs has not been fully elucidated, the regeneration process during aging is severely impaired. In addition, recent studies have shown that a decrease in capillary function reduces the supply of nutrients to muscle tissue, contributing to the decline in muscle mass and functionality during aging [37,38]. In this work, we stained muscle biopsies with anti-PAX7 antibodies and found that sarcopenic patients had a significantly lower number of satellite cells compared with the control group. A reduction in SCs has been associated with defects in the circulatory system [37,38]. Indeed, we detected a decrease in CD31 in skeletal muscle tissue of sarcopenic patients, suggesting a decrease in overall nutrient delivery, including oxygen. However, in contrast to the physiological cellular response to decreased oxygen tension expected in healthy individuals, we found a dramatic reduction in HIF-1α protein levels in sarcopenic patients [39,40]. These results suggest an impairment of the machinery responsible for the cellular response to hypoxic stress, which is crucial for the activation of the muscle regeneration process. [41]. Specifically, the results showed a significant decrease in: (i) VEGF, the main regulator of angiogenesis, which explains the impairment of the circulatory system associated with sarcopenia [37], (ii) GAPDH, the main enzyme involved in the glycolytic pathway and negatively correlated with aging [27], and (iii) WNT7a, a central marker of skeletal muscle hypertrophy [26]. Based on these findings, we envisioned the possibility of developing a novel pharmacological approach to sarcopenia that would counteract the decline of HIF-1α and its target genes. To this end, we isolated primary cultures of human satellite cells from skeletal muscle biopsies of sarcopenic patients and cultured them in the presence of an HIF-1α activator, FG-4592, a drug used in the clinic to treat renal anemia [29]. The results showed that treatment with FG-4592 led to an increase in the expression of HIF-1α target genes, demonstrating reactivation of the hypoxic pathway. Of note, this also led to a decrease in the atrophy marker *MURF1* and promoted upregulation of the stem cell marker *PAX7*. Although these results are still preliminary, they seem to support the notion that pharmacological activation of HIF-1α could counteract the development of sarcopenia by activating muscle regeneration. Clearly, further experiments are needed to understand whether this approach can be further translated to the clinic and further experiments are currently being conducted in our laboratories.

## 4. Materials and Methods

### 4.1. Study Population

From January 2018 to October 2020, a total of 11 non-sarcopenic (aged 25–38 years) and 15 sarcopenic (aged 74–90 years) patients were enrolled from IRCCS Orthopaedic Galeazzi Hospital from those who were scheduled for orthopaedic surgery (hip replacement due to osteoarthritis, proximal femur fractures, and anterior cruciate ligament reconstruction). Written informed consent was obtained from all participants for participation in this study (protocol “Role of the Hypoxia Inducible Factor 1α (HIF-1α) in muscle aging”; Ethics Approval N°: 94/INT/2017 13/07/2017; Clinicaltrial.gov N°: NCT03371134). The study protocol was approved by the San Raffaele Hospital Ethical Review Board. Before surgery, anthropometric examinations, and serological and metabolic analyses were performed in all patients. In addition, the skeletal muscle mass of all sarcopenic patients was quantified by dual-energy X-ray absorptiometry (DXA).

### 4.2. Anthropometric Examination

Standing height and weight were measured to the nearest 0.1 cm and 0.5 kg using a wall-mounted stadiometer and an automated balance. The body mass index (BMI) was calculated using the formula weight (kg)/height^2^ (m^2^).

### 4.3. Blood Sample Analyses

Venous blood was drawn from all patients before surgery and analyzed by the central laboratory of IRCCS Orthopaedic Galeazzi Hospital, which performed preoperative routine blood tests, such as glucose test, total cholesterol, calcium, phosphorus, creatinine. Moreover, HCV, HIV, HBV, and TPHA serological tests were also tested. In case of serological positivity, samples were automatically excluded from the study and discarded.

### 4.4. Whole-Body DXA

The quantitative assessments of regional lean mass, fat mass and bone mass were conducted using dual energy X-ray absorptiometry (DXA) with an Hologic QDR-Discovery W densitometer (Hologic Inc., Bedford, MA, USA) at the IRCCS Istituto Ortopedico Galeazzi in Milano, Italy. Skeletal muscle mass was reported by using the appendicular skeletal muscle index (ASMI) according to the revised European consensus on definition and diagnosis of sarcopenia from the European Working Group on Sarcopenia in Older People (EWGSOP2) [4]. ASMI was used as an index to quantify muscle mass and was obtained by adjusting the overall lean mass of the upper and lower limbs (appendicular skeletal muscle, ASM) for body size (ASM/height^2^). According to EWGSOP2 sarcopenia cut-off points, reduced muscle mass was defined when ASMI was <7.0 kg/m^2^ for men and <5.5 kg/m^2^ for women [4]. Additionally, the whole-body percentage of fat mass was evaluated; similar to ASMI, the overall fat mass was adjusted for body size (fat mass/height^2^) in order to obtain the Fat Mass Index (FMI), which was expressed as kg/m^2^. According to Kelly et al., normal values for FMI were considered in the range between 3 and 6 for men, and between 5 and 9 for women [42].

### 4.5. Skeletal Muscle Tissue Collection

Fragments of tensor fascia latae were harvested from elderly patients during hip arthroplasty, whereas fragments of gracilis muscle and/or semitendinosus muscle were collected from young patients during the tendon harvesting and neoligament preparation phase (standard procedure in anterior cruciate ligament reconstruction surgeries). Tissue samples from young patients were used as a control group. From each patient, 3 sections of skeletal muscle biopsies were taken: one for muscle cell isolation, one for RNA/proteins extraction, and one for immunohistochemistry.

### 4.6. Skeletal Muscle Cells Isolation

Skeletal muscle biopsies for isolation muscle cells were collected in sterile phosphate-buffered saline (PBS, Euroclone) and then dissected in a new Petri dish containing PBS. All subsequent manipulations were performed in a culture hood. Briefly, all visible fat and nerve deposits were removed with forceps. The muscle was placed on a plastic plate and the tissue was minced into a paste with small surgical scissors until no visible muscle deposits remained. The paste was transferred to a tube containing 2% type II collagenase (Thermo Fisher Scientific, Waltham, MA, USA) dissolved in DMEM High Glucose (Dulbecco’s modified Eagle’s medium, Merck) with 1% penicillin-streptomycin (P/S, Euroclone). Approximately 3 mL of collagenase solution was prepared for 1 g of muscle tissue. The mixture was incubated at 37 °C for 60 min on a shaker. After incubation, the homogenate was centrifuged at 300× g for 5 min and the supernatant discarded. The pellet was resuspended in 2 mL of 0.5% trypsin (Thermo Fisher Scientific) and mechanically dissociated using a 5 mL serological pipette. After dissociation, the pellet was allowed to settle by gravity for 5 min, while the supernatant was collected in another tube containing DMEM High Glucose + 10% Horse Serum (HS, Merck). Trypsin dissociation was repeated twice and finally the collected supernatant was filtered in a cell strainer to obtain single cells. A pre-plating phase was then performed to reduce cellular contamination. Specifically, cells were seeded in DMED containing high glucose + 10% horse serum for 2 h. After pre-plating, cells were harvested, centrifuged at 500× g for 10 min, resuspended in growth medium (GM) containing Nutrient Mixture F-10 Ham (Merck), 0.5 mg/mL Bovine Serum Albumin (BSA, Merck), 0.5 mg/mL Fetuin (Merck), 0.39 μg/mL Dexamethasone (Merck), 10 ng/mL Epidermal Growth Factor (EGF, Merck), 0.05 mg/mL Insulin (Merck), 3 mg/mL Glucose (Merck), 15% siero Fetal Bovine Serum (FBS, Merck), 1% Penicillin/Streptomycin (P/S, Merck), and incubated for the following 2 days without changing the medium [43].

### 4.7. TRIzol RNA and Protein Extraction

Skeletal muscle biopsies for RNA/Protein extraction were collected in RNA Later (Thermo Fisher Scientific). TRIzol reagent (Thermo Fisher Scientific) was added to 50 mg of skeletal muscle tissue, which was minced and homogenized using a tissue lyser (Thermo Fisher Scientific). The samples were shaken with tungsten carbide beads (3 mm diameter, Thermo Fisher Scientific) at 900 rpm for 2 min. Then the samples were centrifuged at 12,000× *g* for 5 min at 4 °C, and the clear supernatant was transferred to a new tube. Next, 0.2 mL of chloroform (Merck) was added to the suspension, which was then shaken for 15 s. The mixture was centrifuged at 14,000× *g* for 15 min at 4 °C after incubation at room temperature for 5 min to allow phase separation. The top layer was transferred to a new 2 mL tube and used for RNA extraction, while the bottom layer of phenol was used for protein precipitation. Briefly, RNA was isolated by adding 0.5 mL isopropanol (Merck) to the aqueous phase, incubating at 4 °C, and centrifuging at 12,000× *g* for 10 min at 4 °C. Then, the RNA was washed in 1 mL of 75% ethanol and centrifuged at 7500× *g* for 5 min at 4 °C. Finally, the supernatant was discarded and the RNA pellet was air dried for 15 min, and then resuspended in 50 µL RNase-free water (Thermo Fisher Scientific) containing 0.1 mM EDTA (Merck), and stored at −80 °C. Prior to use, RNA was purified using the ReliaPrepTM RNA Miniprep System (Promega) and transcribed into cDNA using the iScript cDNA Synthesis Kit (BioRad) according to the manufacturer’s instructions. 

To extract the proteins, 0.3 mL of 100% ethanol was added to the interphase/phenol phase, mixed by inverting the tube several times, and incubated for 3 min. After centrifugation, the pellet containing DNA was discarded, while the phenol-ethanol supernatant was transferred to a new tube. To pellet the proteins, 1.5 mL of isopropanol was added to the phenol-ethanol supernatant, incubated for 10 min, and centrifuged for 10 min at 12,000× *g* at 4 °C. The protein pellet was then washed with f 0.3 M guanidine hydrochloride (Merck) in 95% ethanol and air dried. Finally, 1% sodium dodecyl sulfate (SDS, Merck) was added to dissolve the protein pellet, and the solution was stored at −20 °C. Total protein content was determined using the BCA Protein Assay Kit (Pierce) according to the manufacturer’s instructions.

### 4.8. Gene Expression by Real-Time Quantitative PCR (qPCR)

Quantitative PCR was performed on 10 ng of cDNA template, 0.2 μM primers, and GoTaq^®^ qPCR Master Mix (Promega) in 20 μL final volume using a StepOnePlus^®^ Real-Time PCR System (Applied Biosystem, Waltham, MA, USA). The following primers were used: *MURF1* forward 5′-CATCAAAAGCATTGTGGAAGC-3′ and reverse 5′-ATGTTCTCAAAGCCCTGC-3′; *VEGF* forward 5′-TGCCCGCTGCTGTCTAAT-3′ and reverse 5′-CCTCGGTCACTCATCTTCAC-3′; *PHD2* forward 5′-TGGGAGTTGCTGTTGAAGTCG-3′ and reverse 5′-CGTGCCGCCTGGAGAAAC-3′; *GAPDH* forward 5′-GAGTCCACTGGCGTCTTCAC-3′ and reverse 5′-GTTCACACCCATGACGAACA-3′; *PAX7* forward 5′-GAAAACCCAGGCATGTTCAG-3′ and reverse 5′-GCGGCTAATCGAACTCACTAA-3′; *S14* forward 5′-GTGTGACTGGTGGGATGAAGG-3′ and reverse 5′-TTGATGTGTAGGGCGGTGATAC-3′. The amplification program consisted of an initial denaturation at 95 °C for three minutes, followed by 40 cycles of 5 s each at 95 °C and 30 s at 57 °C. Relative quantification of target genes was performed in triplicate and calculated using Equation 2^−^^ΔΔ^^Ct^.

### 4.9. Western Blot

Proteins were boiled for 10 min in sample buffer (0.6 g/100 mL Tris, 2 g/100 mL SDS, 10% glycerol, 1% 2-mercaptoethanol, pH 6.8) and loaded into a 10% SDS-PAGE gel, then transferred to a nitrocellulose membrane (Trans-blot, Bio-Rad Laboratories, Hercules, CA, USA) by electroblotting. The nitrocellulose membranes were incubated with a blocking solution containing 5% (*w*/*v*) nonfat dry milk or 5% (*w*/*v*) BSA (Merck) in Tris buffer saline with 0.1% Tween 20 (TBS-T) for 1 h. Blots were incubated for 2 h at room temperature with the following primary antibodies: mouse monoclonal anti-MURF1, 1:11,000 (Santa Cruz Biotechnology, Dallas, TX, USA), rabbit monoclonal anti-HIF-1α, 1:1000 dilution (clone D2U3T, Cell Signaling Technology, Danvers, MA, USA), rabbit polyclonal anti-WNT7a, 1:1000 dilution (abcam), anti-PHD2, 1:1000 dilution (Cell Signaling Technology), anti-GAPDH, 1:11,000 (Cell Signaling Technology), mouse monoclonal anti-VEGF, 1;1000 (Santa Cruz Biotechnology). Total transferred proteins were used to normalize human proteins using the REVERT Total Protein Stain Kit (LI-COR Biotechnology, Lincoln, NE, USA) according to the manufacturer’s instructions. Membranes were washed three times for 10 min with TBS-T and then incubated with the appropriate anti-mouse, anti-rabbit, or anti-goat HRP-conjugated secondary antibodies (Dako, Agilent Technologies, Santa Clara, CA, USA) at a dilution of 1:2000 for 1 h at room temperature. After washing three times for 10 min with TBS-T, immunoreactive bands were visualized with Enhanced Chemiluminescence Detection Kit reagents (ECL Advance, GeHealthcare, Chicago, IL, USA) according to the manufacturer’s instructions. Optical density was measured with Image Studio TM Lite software (LI-COR Biotechnology).

### 4.10. Immunohistochemistry and Quantification

Human muscle tissue was harvested in formalin, fixed, paraffin embedded and sections were prepared as previously described [44]. Formalin-fixed paraffin-embedded consecutive sections (3 µm thickness) were dewaxed, hydrated by graded decrease alcohol series and stained for histological analysis. The following antibodies were used for immunohistochemical staining (IHC): mouse anti-MURF1, dilution 1:100 (C11, Santa Cruz Biotechnology), mouse anti-PAX7, dilution 1:100 (Developmental Studies Hybridoma Bank, Iowa, IA, USA), rabbit anti-CD31, dilution 1:20 (LSBio, Seattle, Washington, USA). Nuclei were counterstained by hematoxyling according to standard protocol (Mayer’s Hematoxylin, #05-06002/L, Bio Optica, Milano, Italy). Representative images were acquired using a THINDER Imager 3D Tissue microscope (Leica, Wetzlar, Germany) with LAS X Navigator software. The entire slide was scanned at 20× magnification to select CD31 and MurF1 hotspots within five random circular grids of 100 μm diameter. Each CD31-and MurF1-positive signal was manually counted. To quantify PAX7 positive cells, 15 random fields were evaluated at 40× magnification.

### 4.11. Statistical Analysis

Data were described as means ± SD. The nonparametric Mann-Whitney test was used to determine statistical significance using GraphPad Prism 9 software. *p* values of less than 0.05 were considered to be significant. All error bars represent the standard deviation of the mean. Pearson’s correlation was used to evaluate significant associations between MurF1, PAX7 and CD31 expression with sarcopenic condition.

## Data Availability

The raw data supporting the conclusions of this manuscript will be made available by the authors, without undue reservation, to any qualified researcher.

## Supplementary Materials

The following supporting information can be downloaded at: https://www.mdpi.com/article/10.3390/ijms23137114/s1.

## References

[1] United Nations Department of Economic and Social Affairs, Population Division (2020). World Population Ageing 2020 Highlights: Living Arrangements of Older Persons.

[2] Shen Y., Liu D., Li S., He Y., Tan F., Sun X., Li D., Xia X., Hao Q. (2022). Effects of Exercise on Patients Important Outcomes in Older People With Sarcopenia: An Umbrella Review of Meta-Analyses of Randomized Controlled Trials. Front. Med..

[3] Martone A.M., Marzetti E., Calvani R., Picca A., Tosato M., Santoro L., Di Giorgio A., Nesci A., Sisto A., Santoliquido A. (2017). Exercise and Protein Intake: A Synergistic Approach against Sarcopenia. BioMed Res. Int..

[4] Cruz-Jentoft A.J., Bahat G., Bauer J., Boirie Y., Bruyere O., Cederholm T., Cooper C., Landi F., Rolland Y., Sayer A.A. (2019). Sarcopenia: Revised European consensus on definition and diagnosis. Age Ageing.

[5] He N., Zhang Y., Zhang L., Zhang S., Ye H. (2021). Relationship Between Sarcopenia and Cardiovascular Diseases in the Elderly: An Overview. Front. Cardiovasc. Med..

[6] Lena A., Anker M.S., Springer J. (2020). Muscle Wasting and Sarcopenia in Heart Failure-The Current State of Science. Int. J. Mol. Sci..

[7] Han P., Yu H., Ma Y., Kang L., Fu L., Jia L., Chen X., Yu X., Hou L., Wang L. (2017). The increased risk of sarcopenia in patients with cardiovascular risk factors in Suburb-Dwelling older Chinese using the AWGS definition. Sci. Rep..

[8] Frontera W.R., Suh D., Krivickas L.S., Hughes V.A., Goldstein R., Roubenoff R. (2000). Skeletal muscle fiber quality in older men and women. Am. J. Physiol. Cell Physiol..

[9] Rotini A., Martinez-Sarra E., Duelen R., Costamagna D., Di Filippo E.S., Giacomazzi G., Grosemans H., Fulle S., Sampaolesi M. (2018). Aging affects the in vivo regenerative potential of human mesoangioblasts. Aging Cell.

[10] Del Campo A., Contreras-Hernandez I., Castro-Sepulveda M., Campos C.A., Figueroa R., Tevy M.F., Eisner V., Casas M., Jaimovich E. (2018). Muscle function decline and mitochondria changes in middle age precede sarcopenia in mice. Aging.

[11] Cataldi A., Di Giulio C. (2009). “Oxygen supply” as modulator of aging processes: Hypoxia and hyperoxia models for aging studies. Curr. Aging Sci..

[12] Sakuma K., Yamaguchi A. (2012). Sarcopenia and age-related endocrine function. Int. J. Endocrinol..

[13] Visser M., Pahor M., Taaffe D.R., Goodpaster B.H., Simonsick E.M., Newman A.B., Nevitt M., Harris T.B. (2002). Relationship of interleukin-6 and tumor necrosis factor-alpha with muscle mass and muscle strength in elderly men and women: The Health ABC Study. J. Gerontol. Ser. A Biol. Sci. Med. Sci..

[14] Frenkel-Denkberg G., Gershon D., Levy A.P. (1999). The function of hypoxia-inducible factor 1 (HIF-1) is impaired in senescent mice. FEBS Lett..

[15] Katschinski D.M. (2006). Is there a molecular connection between hypoxia and aging?. Exp. Gerontol..

[16] Rhoads R.P., Flann K.L., Cardinal T.R., Rathbone C.R., Liu X., Allen R.E. (2013). Satellite cells isolated from aged or dystrophic muscle exhibit a reduced capacity to promote angiogenesis in vitro. Biochem. Biophys. Res. Commun..

[17] Di Giulio C., Bianchi G., Cacchio M., Artese L., Rapino C., Macri M.A., Di Ilio C. (2005). Oxygen and life span: Chronic hypoxia as a model for studying HIF-1alpha, VEGF and NOS during aging. Respir. Physiol. Neurobiol..

[18] Bodine S.C., Latres E., Baumhueter S., Lai V.K., Nunez L., Clarke B.A., Poueymirou W.T., Panaro F.J., Na E., Dharmarajan K. (2001). Identification of ubiquitin ligases required for skeletal muscle atrophy. Science.

[19] Seale P., Sabourin L.A., Girgis-Gabardo A., Mansouri A., Gruss P., Rudnicki M.A. (2000). Pax7 is required for the specification of myogenic satellite cells. Cell.

[20] Parizkova J., Eiselt E., Sprynarova S., Wachtlova M. (1971). Body composition, aerobic capacity, and density of muscle capillaries in young and old men. J. Appl. Physiol..

[21] DeLisser H.M., Christofidou-Solomidou M., Strieter R.M., Burdick M.D., Robinson C.S., Wexler R.S., Kerr J.S., Garlanda C., Merwin J.R., Madri J.A. (1997). Involvement of endothelial PECAM-1/CD31 in angiogenesis. Am. J. Pathol..

[22] Krock B.L., Skuli N., Simon M.C. (2011). Hypoxia-induced angiogenesis: Good and evil. Genes Cancer.

[23] Berra E., Benizri E., Ginouves A., Volmat V., Roux D., Pouyssegur J. (2003). HIF prolyl-hydroxylase 2 is the key oxygen sensor setting low steady-state levels of HIF-1alpha in normoxia. EMBO J..

[24] Cirillo F., Resmini G., Angelino E., Ferrara M., Tarantino A., Piccoli M., Rota P., Ghiroldi G., Monasky M.M., Ciconte G. (2020). HIF-1α Directly Controls WNT7A Expression During Myogenesis. Front. Cell Dev. Biol..

[25] Higashimura Y., Nakajima Y., Yamaji R., Harada N., Shibasaki F., Nakano Y., Inui H. (2011). Up-regulation of glyceraldehyde-3-phosphate dehydrogenase gene expression by HIF-1 activity depending on Sp1 in hypoxic breast cancer cells. Arch. Biochem. Biophys..

[26] von Maltzahn J., Bentzinger C.F., Rudnicki M.A. (2011). Wnt7a-Fzd7 signalling directly activates the Akt/mTOR anabolic growth pathway in skeletal muscle. Nat. Cell Biol..

[27] Vigelso A., Dybboe R., Hansen C.N., Dela F., Helge J.W., Guadalupe Grau A. (2015). GAPDH and beta-actin protein decreases with aging, making Stain-Free technology a superior loading control in Western blotting of human skeletal muscle. J. Appl. Physiol..

[28] Lin C., McGough R., Aswad B., Block J.A., Terek R. (2004). Hypoxia induces HIF-1alpha and VEGF expression in chondrosarcoma cells and chondrocytes. J. Orthop. Res..

[29] Maxwell P.H., Eckardt K.U. (2016). HIF prolyl hydroxylase inhibitors for the treatment of renal Nat. Rev. Nephrol..

[30] Chen S., Sang N. (2016). Hypoxia-Inducible Factor-1: A Critical Player in the Survival Strategy of Stressed Cells. J. Cell. Biochem..

[31] Cirillo F., Resmini G., Ghiroldi A., Piccoli M., Bergante S., Tettamanti G., Anastasia L. (2017). Activation of the hypoxia-inducible factor 1alpha promotes myogenesis through the noncanonical Wnt pathway, leading to hypertrophic myotubes. FASEB J..

[32] Verdijk L.B., Koopman R., Schaart G., Meijer K., Savelberg H.H., van Loon L.J. (2007). Satellite cell content is specifically reduced in type II skeletal muscle fibers in the elderly. Am. J. Physiol. Endocrinol. Metab..

[33] Brack A.S., Bildsoe H., Hughes S.M. (2005). Evidence that satellite cell decrement contributes to preferential decline in nuclear number from large fibres during murine age-related muscle atrophy. J. Cell Sci..

[34] Chen W., Datzkiw D., Rudnicki M.A. (2020). Satellite cells in ageing: Use it or lose it. Open Biol..

[35] van der Meer S.F., Jaspers R.T., Jones D.A., Degens H. (2011). Time-course of changes in the myonuclear domain during denervation in young-adult and old rat gastrocnemius muscle. Muscle Nerve.

[36] Schafer R., Zweyer M., Knauf U., Mundegar R.R., Wernig A. (2005). The ontogeny of soleus muscles in mdx and wild type mice. Neuromuscul. Disord..

[37] Landers-Ramos R.Q., Prior S.J. (2018). The Microvasculature and Skeletal Muscle Health in Aging. Exerc. Sport Sci. Rev..

[38] Nederveen J.P., Joanisse S., Snijders T., Ivankovic V., Baker S.K., Phillips S.M., Parise G. (2016). Skeletal muscle satellite cells are located at a closer proximity to capillaries in healthy young compared with older men. J. Cachexia Sarcopenia Muscle.

[39] Kim W., Kaelin W.G. (2003). The von Hippel-Lindau tumor suppressor protein: New insights into oxygen sensing and cancer. Curr. Opin. Genet. Dev..

[40] Wang G.L., Jiang B.H., Rue E.A., Semenza G.L. (1995). Hypoxia-inducible factor 1 is a basic-helix-loop-helix-PAS heterodimer regulated by cellular O2 tension. Proc. Natl. Acad. Sci. USA.

[41] Pircher T., Wackerhage H., Aszodi A., Kammerlander C., Bocker W., Saller M.M. (2021). Hypoxic Signaling in Skeletal Muscle Maintenance and Regeneration: A Systematic Review. Front. Physiol..

[42] Kelly T.L., Wilson K.E., Heymsfield S.B. (2009). Dual energy X-Ray absorptiometry body composition reference values from NHANES. PLoS ONE.

[43] Cardani R., Baldassa S., Botta A., Rinaldi F., Novelli G., Mancinelli E., Meola G. (2009). Ribonuclear inclusions and MBNL1 nuclear sequestration do not affect myoblast differentiation but alter gene splicing in myotonic dystrophy type 2. Neuromuscul. Disord..

[44] Zaccagnini G., Palmisano A., Canu T., Maimone B., Lo Russo F.M., Ambrogi F., Gaetano C., De Cobelli F., Del Maschio A., Esposito A. (2015). Magnetic Resonance Imaging Allows the Evaluation of Tissue Damage and Regeneration in a Mouse Model of Critical Limb Ischemia. PLoS ONE.

