# Supplementary figures and images for: Human Sarcopenic Myoblasts Can Be Rescued by Pharmacological Reactivation of HIF-1α
Source: Int J Mol Sci. 2022 Jun 26;23(13):7114. doi: 10.3390/ijms23137114 (PMC9267002; doi:10.3390/ijms23137114)

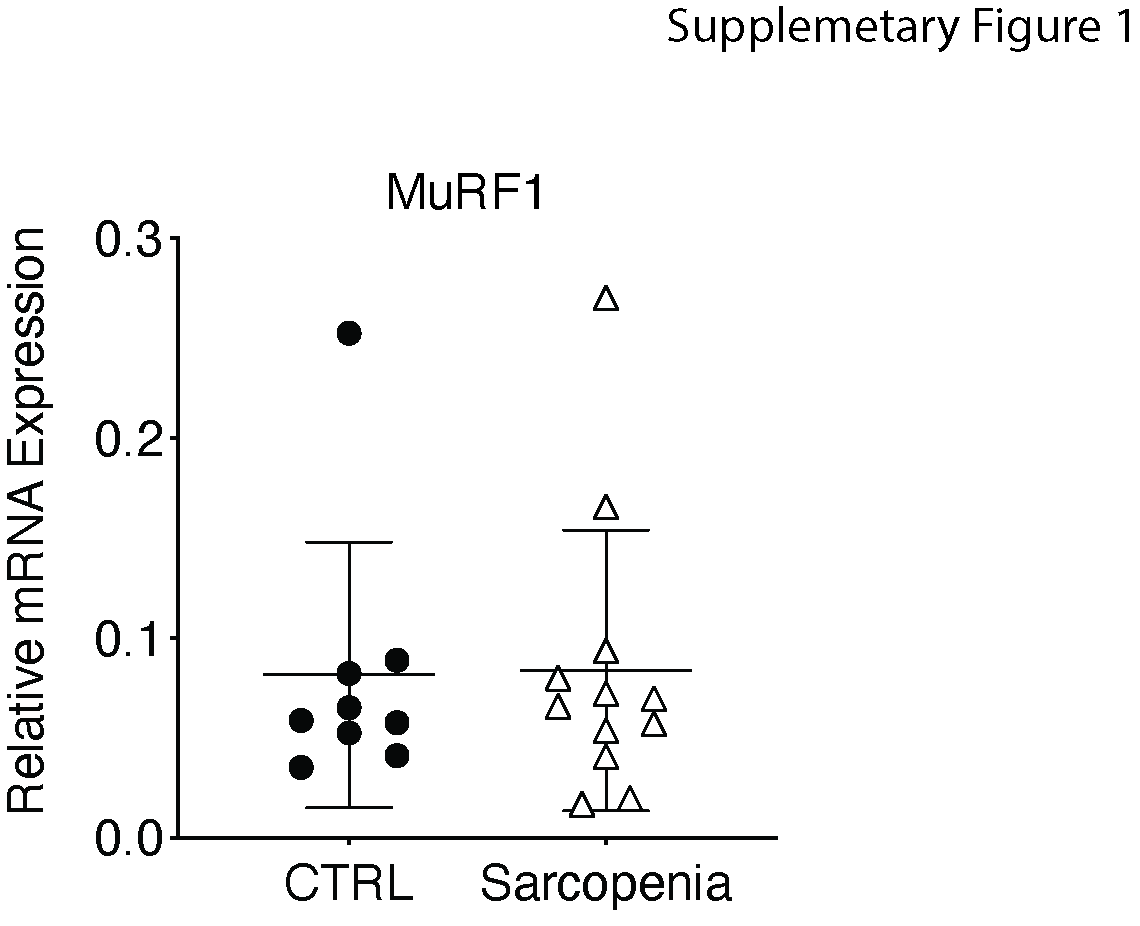

Supplement: Supplementary file 1 [file ijms-23-07114-s001.zip › Suppl 1.tif]
